# Supplementary figures and images for: The importance of making testable predictions: A cautionary tale
Source: PLoS One. 2020 Dec 8;15(12):e0236541. doi: 10.1371/journal.pone.0236541 (PMC7723288; doi:10.1371/journal.pone.0236541)

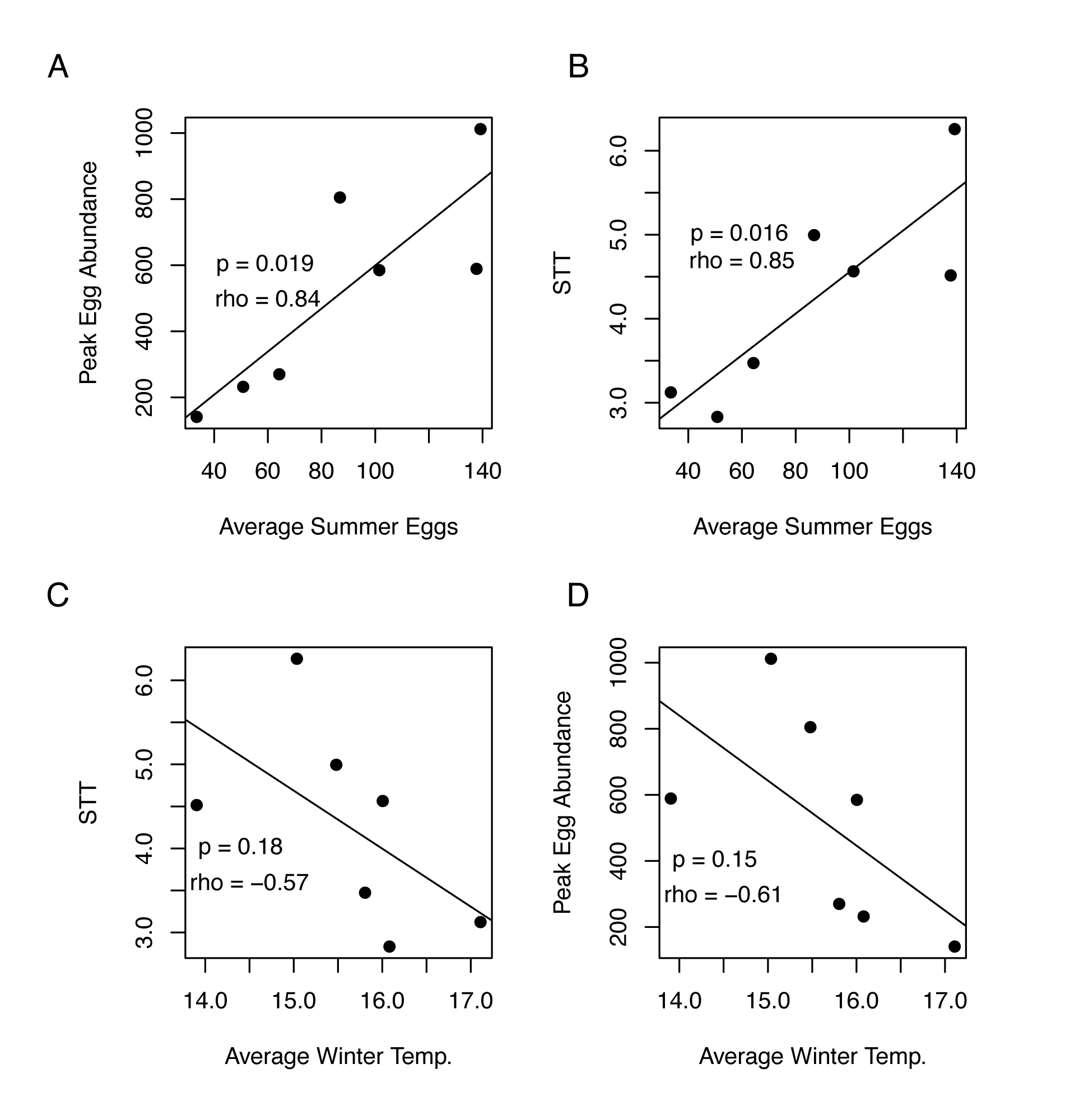

Supplement: S1 Fig — The strongest correlation we found was between STT and peak egg abundance (maximum correlation of 0.98). However, as found by [15], a strong, negative correlation exists between average winter temperatures and average summer egg abundance. Not surprisingly, there is also a strong correlation between peak egg abundance and average egg abundance for a given summer (A). Due to transitivity, there is also a strong correlation between average summer eggs and spring temperature triggers (B). Weaker correlations also exist between average winter temperatures and the finer scale temperature triggers (C) and peak summer egg abundance (D), however these are much weaker relationships (p> 0.1). (TIF) [file pone.0236541.s001.tif]

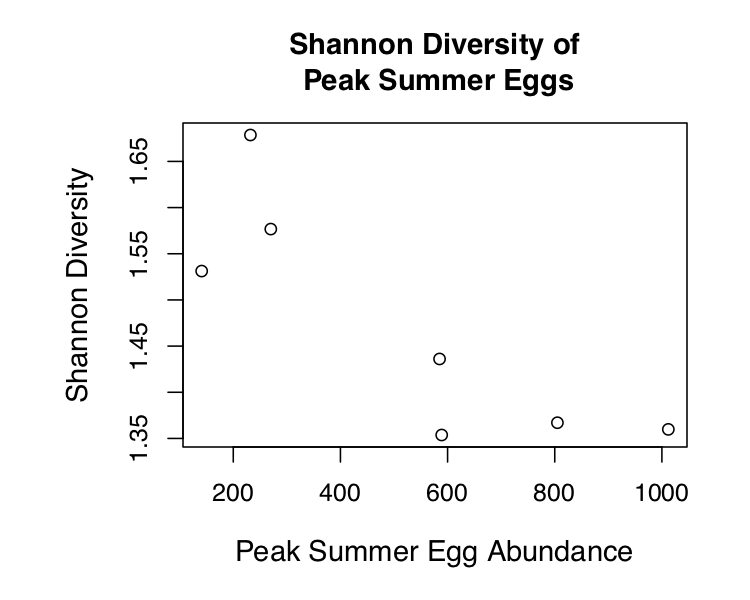

Supplement: S2 Fig — Shannon diversity (base e) appears to be higher for peaks with lower abundance. (TIF) [file pone.0236541.s002.tif]
