# Supplementary material for: The importance of making testable predictions: A cautionary tale
Source: PLoS One. 2020 Dec 8;15(12):e0236541. doi: 10.1371/journal.pone.0236541 (PMC7723288; doi:10.1371/journal.pone.0236541)
Supplement: S1 Table — The proportional contribution that each of the identified species contributes to the annual peak summer egg abundance. The peak samples in each year are dominated by the eggs of a few species, with the dominant species varying from year-to-year. (DOCX) [file pone.0236541.s003.docx]

| S1 Table. Species composition of the peak summer egg abundance samples. The proportional contribution that each of the identified species contributes to the annual peak summer egg abundance. The peak samples in each year are dominated by the eggs of a few species, with the dominant species varying from year-to-year. | | | | | | | | |
| --- | --- | --- | --- | --- | --- | --- | --- | --- |
| *Scientific Name* | *Common Name* | *2013* | *2014* | *2015* | *2016* | *2017* | *2018* | *2019* |
| *Xenistius californiensis* | California salema | 0.668 | 0.041 | 0.010 | 0.108 | 0.741 | 0.030 | 0.01 |
| *Citharichthys stigmaeus* | Speckled sanddab | 0.064 | 0.074 | 0.281 | 0.312 | 0.036 | 0.429 | 0.33 |
| *Oxyjulis californica* | Senorita | 0.176 | 0.721 | 0.010 | 0.344 | 0.050 | 0.000 | 0.00 |
| *Menticirrhus undulatus* | California corbina | 0.011 | 0.008 | 0.479 | 0.102 | 0.000 | 0.157 | 0.06 |
| *Roncador stearnsii* | Spotfin croaker | 0.005 | 0.000 | 0.010 | 0.006 | 0.000 | 0.339 | 0.35 |
| *Seriphus politus* | Queenfish | 0.027 | 0.031 | 0.104 | 0.000 | 0.029 | 0.012 | 0.06 |
| *Halichoeres semicinctus* | Rock wrasse | 0.000 | 0.006 | 0.000 | 0.019 | 0.065 | 0.000 | 0.15 |
| *Paralichthys californicus* | California halibut | 0.000 | 0.004 | 0.073 | 0.000 | 0.000 | 0.002 | 0.00 |
| *Paralabrax clathratus* | Kelp bass | 0.000 | 0.017 | 0.000 | 0.038 | 0.007 | 0.000 | 0.00 |
| *Umbrina roncador* | Yellowfin croaker | 0.000 | 0.004 | 0.010 | 0.019 | 0.022 | 0.005 | 0.00 |
| *Scomber japonicus* | Chub mackerel | 0.027 | 0.017 | 0.000 | 0.006 | 0.007 | 0.000 | 0.00 |
| *Semicossyphus pulcher* | Sheephead | 0.005 | 0.016 | 0.000 | 0.006 | 0.022 | 0.000 | 0.00 |
| *Trachurus symmetricus* | Pacific jack mackerel | 0.000 | 0.027 | 0.000 | 0.000 | 0.000 | 0.000 | 0.00 |
| *Paralabrax nebulifer* | Barred sand bass | 0.000 | 0.000 | 0.010 | 0.000 | 0.014 | 0.000 | 0.00 |
| *Anisotremus davidsonii* | Xantic sargo | 0.000 | 0.021 | 0.000 | 0.000 | 0.000 | 0.000 | 0.00 |
| *Hermosilla azurea* | Zebra perch sea chub | 0.000 | 0.000 | 0.000 | 0.013 | 0.000 | 0.002 | 0.00 |
| *Citharichthys sordidus* | Pacific sanddab | 0.005 | 0.001 | 0.000 | 0.000 | 0.000 | 0.002 | 0.01 |
| *Cynoscion parvipinnis* | Shortfin weakfish | 0.000 | 0.000 | 0.000 | 0.006 | 0.000 | 0.007 | 0.00 |
| *Genyonemus lineatus* | White croaker | 0.000 | 0.001 | 0.000 | 0.000 | 0.007 | 0.005 | 0.00 |
| *Scorpaena guttata* | Calfornia scorpion fish | 0.000 | 0.000 | 0.000 | 0.013 | 0.000 | 0.000 | 0.00 |
| *Symphurus atricaudus* | California tonguefish | 0.000 | 0.000 | 0.010 | 0.000 | 0.000 | 0.000 | 0.00 |
| *Cheilotrema saturnum* | Black croaker | 0.000 | 0.003 | 0.000 | 0.006 | 0.000 | 0.000 | 0.00 |
| *Paralabrax maculatofasciatus* | Spotted sand bass | 0.000 | 0.000 | 0.000 | 0.000 | 0.000 | 0.000 | 0.01 |
| *Citharichthys xanthostigma/sordidus* | Pacific/Longfin sanddab | 0.005 | 0.001 | 0.000 | 0.000 | 0.000 | 0.000 | 0.00 |
| *Chilara taylori* | Spotted cusk eel | 0.005 | 0.000 | 0.000 | 0.000 | 0.000 | 0.000 | 0.00 |
| *Atractoscion nobilis* | White seabass | 0.000 | 0.004 | 0.000 | 0.000 | 0.000 | 0.000 | 0.00 |
| *Xystreurys liolepis* | Fantail sole | 0.000 | 0.000 | 0.000 | 0.000 | 0.000 | 0.000 | 0.00 |
| *Hypsopsetta guttulata* | Diamond turbot | 0.000 | 0.000 | 0.000 | 0.000 | 0.000 | 0.002 | 0.00 |
| *Fodiator acutus* | Sharpchin flyingfish | 0.000 | 0.000 | 0.000 | 0.000 | 0.000 | 0.002 | 0.00 |
| *Strongylura exilis* | California needlefish | 0.000 | 0.000 | 0.000 | 0.000 | 0.000 | 0.002 | 0.00 |
| *Sardinops sagax* | Pacific sardine | 0.000 | 0.001 | 0.000 | 0.000 | 0.000 | 0.000 | 0.00 |
