# Supplementary material for: The importance of making testable predictions: A cautionary tale
Source: PLoS One. 2020 Dec 8;15(12):e0236541. doi: 10.1371/journal.pone.0236541 (PMC7723288; doi:10.1371/journal.pone.0236541)
Supplement: S3 Table — A list of all 46 species identified in the samples from Scripps Pier from 2013–2019 and the proportion of samples they were present in within each year. The sampling effort by year is as follows: 2013 = 161, 2014 = 84, 2015 = 51, 2016 = 52, 2017 = 48, 2018 = 75, 2019 = 65. (DOCX) [file pone.0236541.s005.docx]

| S3 Table. Scripps Pier Species Frequency 2013 – 2019. A list of all 46 species identified in the samples from Scripps Pier from 2013 – 2019 and the proportion of samples they were present in within each year. The sampling effort by year is as follows: 2013 = 72, 2014 = 35, 2015 = 17, 2016 = 18, 2017 = 17, 2018 = 38, 2019 = 28. | | | | | | | | |
| --- | --- | --- | --- | --- | --- | --- | --- | --- |
| *Scientific Name* | *Common Name* | *2013* | *2014* | *2015* | *2016* | *2017* | *2018* | *2019* |
| *Citharichthys stigmaeus* | Speckled sanddab | 0.919 | 0.762 | 0.490 | 0.788 | 0.979 | 0.880 | 0.908 |
| *Oxyjulis californica* | Senorita | 0.553 | 0.500 | 0.451 | 0.385 | 0.479 | 0.387 | 0.292 |
| *Sardinops sagax* | Pacific sardine | 0.112 | 0.143 | 0.059 | 0.019 | 0.125 | 0.293 | 0.062 |
| *Xenistius californiensis* | Californian salema | 0.149 | 0.202 | 0.098 | 0.058 | 0.146 | 0.413 | 0.138 |
| *Engraulis mordax* | Anchovy | 0.174 | 0.155 | 0.020 | 0.058 | 0.271 | 0.293 | 0.169 |
| *Halichoeres semicinctus* | Rock wrasse | 0.261 | 0.238 | 0.118 | 0.115 | 0.312 | 0.480 | 0.338 |
| *Menticirrhus undulatus* | California corbina | 0.217 | 0.214 | 0.078 | 0.135 | 0.208 | 0.360 | 0.185 |
| *Roncador stearnsii* | Spotfin croaker | 0.205 | 0.107 | 0.078 | 0.115 | 0.250 | 0.307 | 0.200 |
| *Citharichthys sordidus* | Pacific sanddab | 0.497 | 0.238 | 0.059 | 0.077 | 0.271 | 0.227 | 0.277 |
| *Citharichthys xanthostigma/sordidus* | Pacific/Longfin sanddab | 0.547 | 0.226 | 0.000 | 0.000 | 0.104 | 0.120 | 0.215 |
| *Paralichthys californicus* | California halibut | 0.342 | 0.429 | 0.078 | 0.096 | 0.396 | 0.400 | 0.538 |
| *Seriphus politus* | Queenfish | 0.130 | 0.143 | 0.020 | 0.058 | 0.271 | 0.253 | 0.200 |
| *Paralabrax clathratus* | Kelp bass | 0.124 | 0.202 | 0.059 | 0.058 | 0.062 | 0.120 | 0.092 |
| *Semicossyphus pulcher* | Sheephead | 0.168 | 0.143 | 0.098 | 0.115 | 0.188 | 0.147 | 0.092 |
| *Trachurus symmetricus* | Pacific jack mackerel | 0.106 | 0.036 | 0.000 | 0.000 | 0.062 | 0.000 | 0.000 |
| *Genyonemus lineatus* | White croaker | 0.056 | 0.060 | 0.000 | 0.077 | 0.167 | 0.120 | 0.031 |
| *Umbrina roncador* | Yellowfin croaker | 0.025 | 0.012 | 0.059 | 0.038 | 0.083 | 0.213 | 0.046 |
| *Hypsopsetta guttulata* | Diamond turbot | 0.217 | 0.131 | 0.078 | 0.096 | 0.042 | 0.093 | 0.092 |
| *Scomber japonicus* | Chub mackerel | 0.056 | 0.048 | 0.039 | 0.058 | 0.125 | 0.107 | 0.015 |
| *Citharichthys xanthostigma* | Longfin sanddab | 0.174 | 0.060 | 0.000 | 0.038 | 0.062 | 0.067 | 0.246 |
| *Anisotremus davidsonii* | Xantic sargo | 0.019 | 0.071 | 0.020 | 0.019 | 0.042 | 0.200 | 0.000 |
| *Symphurus atricaudus* | California tonguefish | 0.031 | 0.048 | 0.059 | 0.038 | 0.125 | 0.133 | 0.077 |
| *Cheilotrema saturnum* | Black croaker | 0.087 | 0.095 | 0.039 | 0.019 | 0.021 | 0.000 | 0.015 |
| *Cynoscion parvipinnis* | Shortfin weakfish | 0.062 | 0.024 | 0.020 | 0.019 | 0.021 | 0.027 | 0.031 |
| *Paralabrax nebulifer* | Barred sand bass | 0.043 | 0.048 | 0.020 | 0.000 | 0.083 | 0.053 | 0.031 |
| *Peprilus simillimus* | Pacific pompano | 0.068 | 0.048 | 0.000 | 0.000 | 0.000 | 0.013 | 0.000 |
| *Xystreurys liolepis* | Fantail sole | 0.006 | 0.024 | 0.000 | 0.019 | 0.062 | 0.040 | 0.046 |
| *Pleuronichthys coenosus* | C.O. sole | 0.019 | 0.000 | 0.000 | 0.019 | 0.021 | 0.160 | 0.046 |
| *Chilara taylori* | Spotted cusk eel | 0.062 | 0.048 | 0.000 | 0.019 | 0.042 | 0.013 | 0.000 |
| *Paralabrax maculatofasciatus* | Spotted sand bass | 0.012 | 0.000 | 0.000 | 0.000 | 0.083 | 0.080 | 0.046 |
| *Hermosilla azurea* | Zebra perch sea chub | 0.006 | 0.000 | 0.000 | 0.019 | 0.042 | 0.120 | 0.031 |
| *Atractoscion nobilis* | White seabass | 0.006 | 0.083 | 0.000 | 0.000 | 0.000 | 0.000 | 0.000 |
| *Pleuronichthys verticalis* | Hornyhead turbot | 0.025 | 0.012 | 0.000 | 0.019 | 0.021 | 0.053 | 0.031 |
| *Girella nigricans* | Opaleye | 0.012 | 0.000 | 0.000 | 0.000 | 0.000 | 0.067 | 0.000 |
| *Caulolatilus princeps* | Ocean whitefish | 0.012 | 0.012 | 0.000 | 0.000 | 0.000 | 0.013 | 0.000 |
| *Hypsoblennius jenkinsi* | Mussel blenny | 0.012 | 0.000 | 0.000 | 0.000 | 0.000 | 0.027 | 0.015 |
| *Sphyraena argentea* | Pacific baracuda | 0.006 | 0.024 | 0.000 | 0.000 | 0.000 | 0.000 | 0.000 |
| *Seriola lalandi* | Yellowtail amberjack | 0.000 | 0.012 | 0.020 | 0.000 | 0.000 | 0.027 | 0.000 |
| *Mugil cephalus* | Flathead grey mullet | 0.000 | 0.000 | 0.020 | 0.000 | 0.021 | 0.000 | 0.015 |
| *Lycodes pacificus* | Blackbelly eelpout | 0.000 | 0.000 | 0.000 | 0.019 | 0.000 | 0.000 | 0.000 |
| *Ophidion scrippsae* | Basketweave cusk eel | 0.000 | 0.000 | 0.000 | 0.000 | 0.021 | 0.013 | 0.000 |
| *Scorpaena guttata* | Calfornia scorpion fish | 0.000 | 0.000 | 0.000 | 0.019 | 0.000 | 0.000 | 0.000 |
| *Fodiator acutus* | Sharpchin flyingfish | 0.000 | 0.000 | 0.000 | 0.000 | 0.000 | 0.027 | 0.000 |
| *Etrumeus acuminatus* | Round herring | 0.000 | 0.000 | 0.000 | 0.000 | 0.000 | 0.013 | 0.000 |
| *Citharichthys gordae* | Mimic sanddab | 0.006 | 0.000 | 0.000 | 0.000 | 0.000 | 0.000 | 0.000 |
| *Stereolepis gigas* | Giant sea bass | 0.006 | 0.000 | 0.000 | 0.000 | 0.000 | 0.000 | 0.000 |
| *Strongylura exilis* | California needlefish | 0.000 | 0.000 | 0.000 | 0.000 | 0.000 | 0.013 | 0.000 |
