# Supplementary material for: The importance of making testable predictions: A cautionary tale
Source: PLoS One. 2020 Dec 8;15(12):e0236541. doi: 10.1371/journal.pone.0236541 (PMC7723288; doi:10.1371/journal.pone.0236541)
Supplement: Attachment — Submitted filename: Response to Reviewers_9.01.2020.pdf [file pone.0236541.s006.pdf]

Dear Editor,

Thank you for considering our manuscript 'Temperature triggers provide quantitative predictions of multi-species fish spawning peaks' and suggesting revisions. We are pleased to submit a revised version of our manuscript that we hope you will now find suitable for publication in PLOS ONE.

We have been able to substantially address all of the comments of Reviewer 1 (see below) through the addition of three new supplemental tables that give detailed breakdowns and summaries of the fish egg species observed in our data, as well as short clarifying statements in the Discussion and Methods sections of the manuscript that reference these tables. Reviewer 1 also made a more general suggestion about a potential alteration of focus of the manuscript toward detailed mechanisms, however we feel that: i) adequately addressing the suggested potential new focus is beyond the scope of our current experimental design; and ii) by retaining our current focus we are better able to highlight for the community the unusual properties and potential general significance of our observations independent of specific mechanisms, thereby leaving open this interesting mechanistic angle to future investigations.

In addition to the new supplemental tables, we have also now included an active link to a GitHub repository that contains all of the data and analysis code used for this manuscript. These materials can also be submitted to Dryad if preferred.

We thank the Editor and the Reviewer for their constructive and insightful input that we feel has greatly improved this revised manuscript.

Sincerely,
George Sugihara
(on behalf of the Authors)

Response to Reveiwer 1

Reviewer's comment: *What is the species composition of the egg catch? I am unsure with the statement that eggs are buoyant. Many are not, and it depends on the species.*

The Reviewer raises an important point, and we're sure many people will be interested in the composition of the samples. To address this, we have included two new supplemental tables. The first of these two tables, *Table S2: Scripps Pier Species Abundance 2013–2019*, lists all 46 of the species identified from the sampling at Scripps Pier from 2013 to 2019 and the number of eggs identified as each of these species within each year. The second of these two tables, *Table S3: Scripps Pier Species Frequency 2013–2019,* lists all 46 of the species and the proportion of samples (out of the yearly sampling effort) in which eggs from the species were observed within each year.

With regard to buoyancy, we have added a statement to the manuscript that our methods only effectively sample eggs suspended in the water column; we found very few eggs from species with demersal eggs – those were presumably stirred off the bottom by the net.

Reviewer's comment: *Somewhat related to my previous question, Fig S2 is interesting and it shows decline of egg diversity in relation to peak eggs abundance. This is presumably driven by an increase of dominance of few or a single species. Can you elaborate on the species that dominate the samples, especially when egg abundance increases? At the end of the methods the authors indicate that the morphologically distinct anchovy and sardine eggs were removed, and the rest of the eggs were counted and identified to species using DNA barcoding. Would be great to see the species list.*

We thank the Reviewer for highlighting this opportunity for clarification. We have now included a new supplemental table, *Table S1: Species composition of the peak summer egg abundance samples*, that lists the proportion of the annual summer peak eggs that were identified as each species. This table highlights which species dominate the peak summer egg abundance samples. We have slightly expanded the main text in the discussion of synchrony to accommodate the addition of this table.

Reviewer's comment: *This is a comment/suggestion. I suggest redirecting the focus of the research question toward a more mechanistic relationship between temperature and egg abundance. The author ask whether 'finer-timescale temperature dynamics provide information about finer-timescale fish egg abundance dynamics.' However, the striking relationships that they have uncovered between STT and peak egg abundance, in my view, is still an integrated measure rather than an examination of a finer scale relationship between temperature and eggs. There is still value in this relationship of course, but not of the same type suggested by the author. This analyses reveals potential mechanisms, pointing to the fact that large variations of water temperature during spring, may trigger massive spawning events during summer.*

While we greatly appreciate this suggestion, and would like to be able to pursue it, it is not currently possible to redirect our research question to address the finely resolved details of the mechanistic relationship between temperature and egg abundance. This is because we did not specifically measure a variable to demonstrate *how* temperature is acting to influence fish reproduction. Given that we are measuring a macroscopic output variable, egg abundance, it is difficult to determine whether the STT is having a direct physiological effect on the fish or whether it is related to other more proximate factors that drive increases in egg abundance. These are questions perhaps best answered by experimental manipulation; in the current experimental design of our study we could not discern the finer-scale details of the mechanism at play. The causality detection method (CCM), however, does verify (within these data) that there is a causal link here between temperature and egg abundance insofar as changes in temperature propagate to changes in egg abundance. Moreover, we show that the strength of the relationship identified here is dependent upon fine time scale measurements – both the STT and the peak summer egg abundance are captured through frequent measurements, daily in the case of STT and weekly in the case of peak summer egg abundance. In Figure 3A we showed that in smoothing the daily

temperature datum, we lose the signal between the STT and peak summer egg abundance. Therefore, we focused our research question on fine time scale dynamics that are shown to be essential to this analysis, rather than a mechanistic relationship that we are unable to speak to, given the output variable we measured.
